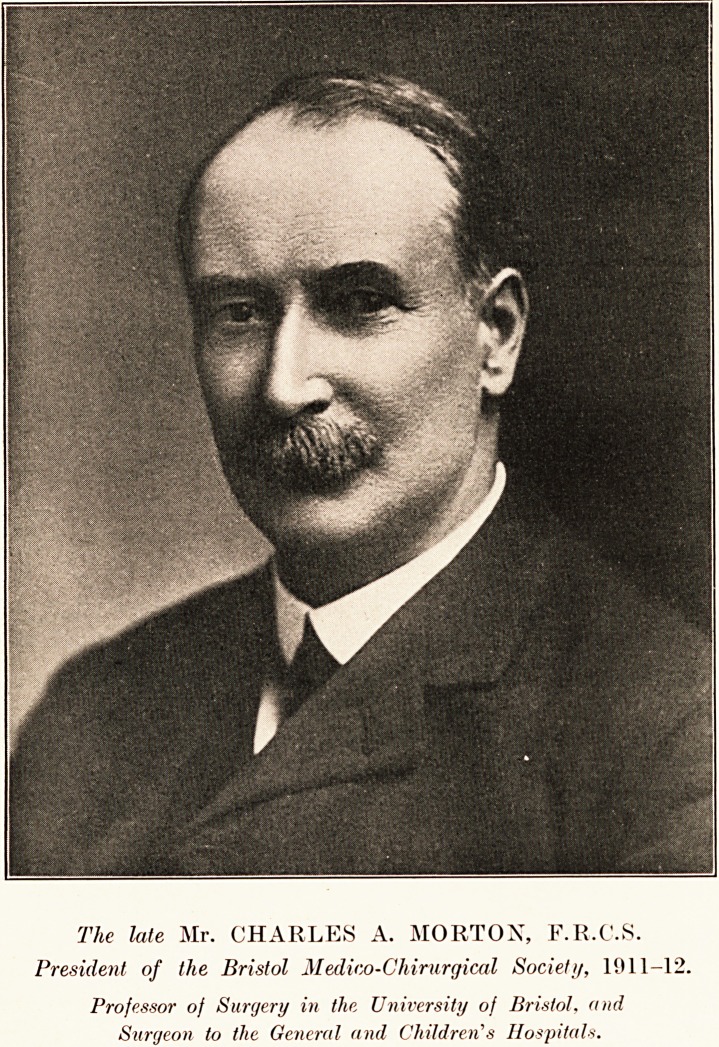# Charles Alexander Morton

**Published:** 1929

**Authors:** 


### Obituary

### CHARLES ALEXANDER MORTON, O.B.E., F.R.C.S.

We regret to report the death of Mr. C. A. Morton, which took place on August 16th during a holiday in Switzerland.

Charles Alexander Morton, known to many generations of students as "Peter," was born in Bristol in 1860, where his father, a surgeon in the H.E.I.C.S., lived after his retirement. He was educated at Clifton, and subsequently at St. Bartholomew's. Mr. Stanford Morton, of ophthalmoscopic fame, who died recently in Clifton, was his half-brother.

His student career was quite distinguished, and many prizes and exhibitions fell to him, including the renowned Brackenbury Medical Scholarship.

After qualifying in 1881 he became House Surgeon at the Stanley Hospital, Liverpool, and then held some house appointments at his own hospital, going on from there to further resident and clinical work at other London hospitals. In 1884 he was appointed House Surgeon at the Cumberland Infirmary, Carlisle.

In 1891, after returning to the city of his birth, he became Registrar to the Bristol General Hospital—he had by then taken his F.R.C.S. Eng.—and, on a vacancy occurring soon after, he was elected Assistant Surgeon.

He retired as Senior Surgeon in 1920. For twenty-five years he was also Surgeon to the Bristol Children's Hospital, and when the amalgamation question was first raised some years ago led the opposition to it.

In the days when it was customary to combine the holding of a hospital appointment with general practice Morton from the first struck out an original line in devoting himself purely to surgery.

In 1897 he became Professor of Surgery in the then University College, continuing his appointment in the new

University that followed it. He resigned this in 1925 on reaching the age limit, and was made an Emeritus Professor.

He was President of the Bristol Medico-Chirurgical Society in 1911. During the war he served in the R.A.M.C. (T.) with the rank of Major, and received the O.B.E. for his work at the Beaufort War Hospital.

In regard to the practical side of surgery, he was a clever and able diagnostician, leaving nothing to chance in his efforts to solve the problems that confronted him. His surgical technique, especially in the preparation of the patient, he pursued with a thoroughness which, though his assistants may often have found it tedious, was prompted by the ideal of doing everything that was possible to ensure the patient's recovery. Both in this and his indifference to fees his splendid altruism at any rate could never be gainsaid.

Morton contributed frequently to medical literature, and his interest in matters of public health and local government drew from him letters on these subjects in the daily press ; in fact, only a few weeks before he died *The Times* published a long letter by him relating to some recent alterations in Poor Law administration. He was for some years a member of the Bristol Board of Guardians.

Sometimes he seemed to those amongst whom he lived as rather a lonely figure, but probably the stimulus and inspiration of personal daily contact that comes to most of us from the camaraderie of our common calling he would get rather from the wide open spaces of hills, moorland and sea of which he was so passionately fond.

It has been told that he once said to a friend in a spirit of frankness not usual with him that he hoped when it was to be that he might die amongst the mountains of Switzerland. If this were so he had his wish, for it was amongst their shadows that he passed away. Whatever place his name will come to hold in the annals of his craft it may be difficult to say, but it is certain that the memory of his kindliness and care will be long enshrined in many humble homes.

*The late* Mr. CHARLES A. MORTON, F.R.C.S.
*President of the Bristol Medico-Chirurgical Society,* 1911–12.
*Professor of Surgery in the University of Bristol, and Surgeon to the General and Children's Hospitals.*

## Figures and Tables

**Figure f1:**